# Feasibility of rapid and automated importation of 3D echocardiographic left ventricular (LV) geometry into a finite element (FEM) analysis model

**DOI:** 10.1186/1475-925X-3-32

**Published:** 2004-10-08

**Authors:** Janko F Verhey, Nadia S Nathan

**Affiliations:** 1 Department of Medical Informatics, University Hospital Goettingen, Robert-Koch-Straße-40, 37075-Göttingen, Germany; 2 Department of Anesthesiology, Brigham and Women's Hospital, 75 Francis Street, Boston, MA 02115, USA

## Abstract

**Background:**

Finite element method (FEM) analysis for intraoperative modeling of the left ventricle (LV) is presently not possible. Since 3D structural data of the LV is now obtainable using standard transesophageal echocardiography (TEE) devices intraoperatively, the present study describes a method to transfer this data into a commercially available FEM analysis system: ABAQUS^©^.

**Methods:**

In this prospective study TomTec LV Analysis TEE^© ^Software was used for semi-automatic endocardial border detection, reconstruction, and volume-rendering of the clinical 3D echocardiographic data. A newly developed software program MVCP FemCoGen^©^, written in Delphi, reformats the TomTec file structures in five patients for use in ABAQUS and allows visualization of regional deformation of the LV.

**Results:**

This study demonstrates that a fully automated importation of 3D TEE data into FEM modeling is feasible and can be efficiently accomplished in the operating room.

**Conclusion:**

For complete intraoperative 3D LV finite element analysis, three input elements are necessary: 1. time-gaited, reality-based structural information, 2. continuous LV pressure and 3. instantaneous tissue elastance. The first of these elements is now available using the methods presented herein.

## Background

Intraoperative TEE is currently available in most cardiac surgical operating rooms. In some centers, intraoperative 3D echocardiography is used to evaluate geometry and to plan surgical interventions prior to LV remodeling surgery. However, quantitation of LV geometry is limited to rather imprecise measures such as ejection fraction. Thus the cardiac surgeon has no sophisticated, immediate, quantitative analysis of the preoperative 3D LV geometry. Intraoperative quantitative analysis of the dynamic behavior of the LV might provide optimal information upon which to base precise patient-specific planning of the surgical intervention, as well as to assess the adequacy of the completed surgical repair.

Because the LV cannot be realistically described by a symmetric mathematical model, the modern approach consists of using a FEM mesh which approximates LV geometry [1] or whole heart geometry [2].

Initial attempts at FEM in the heart have been carried out with 3D segmentation and tracking using sophisticated and expensive cardiac MRI [3]. MRI is impractical in the cardiac surgical operating room and is complicated by the fact that the LV and the papillary muscles are active materials, behaving differently during systole and diastole. An ideal model would provide material properties specific to each patient as first mentioned by McCulloch [4], but untill now patient-specific modeling in the operating room is not been possible.

FEM modeling of 3D intraoperative echo data provides an excellent tool for incorporating material properties, volumetric data and boundary pressures to more accurately record and then to simulate LV dynamic performance. Accurate simulation will be the foundation of surgical planning. The limitation until now in applying FEM intraoperatively has been the technical complexity of this technique. The purpose of this study is to take the first step towards introducing FEM into the operating room environment. The goal is to facilitate transfer of geometric data from 3D ultrasound data set into FEM.

## Methods

After obtaining institutional review board approval, LV images from clinical TEE data sets were obtained in five patients via the midesophageal window using a Philips 5500/7500 or and Acuson Sequoia ultrasound system. After induction of general anesthesia and airway protection, the esophagus was intubated using an omniplane TEE probe. 3D TEE data sets of the LV structures including mitral annulus and leaflets, chordae tendinae, papillary muscles and ventricular wall were obtained using the automated Philips/Acuson acquisition protocol at 10° increment. Images were gaited for both beat-to-beat variability and respiratory motion. In order to facilitate acquisition in the shortest possible timeframe, ventilation was modified to provide a tidal volume of 5*10^-6 ^m^-3 ^kg^-1 ^at a respiratory rate sufficient to maintain end-tidal CO_2 _levels between ~4.4*10^3 ^m kg^-1 ^s^-1 ^and ~5.1*10^3 ^m kg^-1^s^-1^.

**Figure 1:**
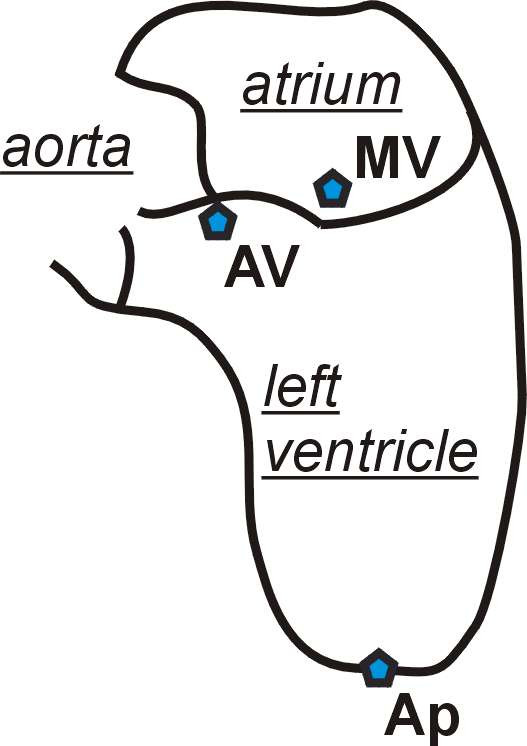
**Scheme of the LV (left ventricle). **Section through left atrium and ventricle shown schematically. In the LV Analysis TomTec TEE program, three landmarks are taken from each second frame per data set. This means that each 10° a frame is taken as the sampling point for the LV Analysis TEE program. AV is aortic valve, MV is mitral valve and Ap is apex.

All images were stripped of patient identifiers. For LV geometry reconstruction, the TomTec LV-Analysis TEE^© ^software module [5] was employed. This software runs on a standard Dell Inspirion laptop computer with Microsoft Windows™ 2000 operating system which imports, analyzes, reports and archives the time-resolved 3D-ultrasound data. The TomTec system automatically detects endocardial borders and produces a 3D shell reconstruction of the LV [5]. It also provides for an analysis of global and regional LV parameters in which a landmark-setting method is used (see Fig. 1).

**Figure 2:**
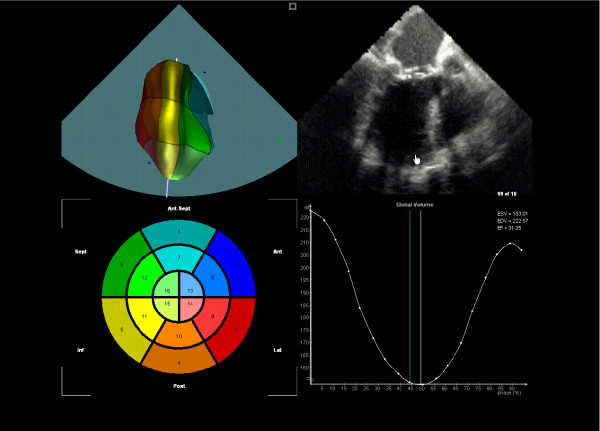
**Screenshot-TomTec. **Screenshot of the workspace of the TomTec LV Analysis TEE program. The LV is segmented using color coding in (c). In (a) the LV model is shown in 3D as calculated from the sampling points set according to Fig. 1. The shadowed plane in (a) indicates the position of the actual original US gray-value frame in 3d as shown in (b). In (d) the volume content is displayed in terms of the actual model step indicating the actual phase with a green line. The screenshot of the actual phase shows the LV model at near systole.

The first landmark was set in the middle of the mitral valve at the level of its annulus. Care was taken to avoid having the mitral valve cusps cross this landmark. Two additional landmarks were placed in the middle of the aortic valve at the level of its annulus and at the endocardial level of the LV apex. With this landmarking procedure, a time-resolved LV geometric analysis with 18 models per heart cycle was obtained (see Fig. 2).

The rendered LV geometry resulting from the TomTec analysis tool was transferred to an ABAQUS input file using software written in Delphi. In this program, the TomTec file structure was reformatted to an ABAQUS system (version 6.3) input file based on standard ABAQUS FEM elements.

ABAQUS creates a time series of LV model files and requires continuous intraventricular pressure and tissue elastance parameters to process the model. For this analysis LV pressure was modeled using wave forms obtained from Columbia University's HeartSim^© ^cardiac simulator [6]. A single tissue elastance parameter was applied. These modeled values were used to demonstrate the concept. Actual values will be needed for accurate simulations.

The time required for each step in this process was recorded for each patient data set.

## Results

**Figure 3:**
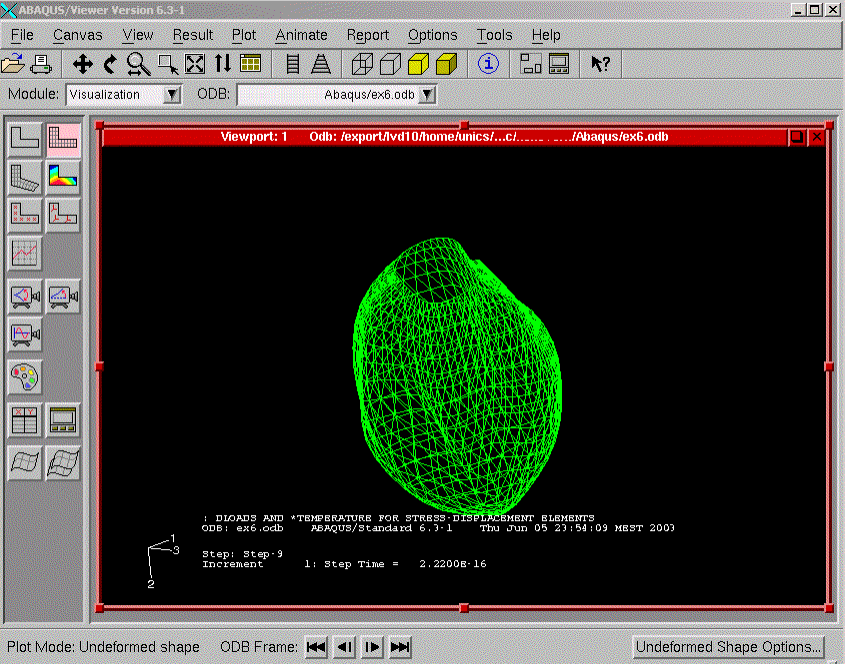
**LV in finite element analysis program. **Left ventricle FEM model in ABAQUS FEM program interface. Shown is the LV in diastole. At the top of the mesh is the aortic valve depicted as a cavity. The LV apex appears at the bottom of the mesh.

**Figure 4:**
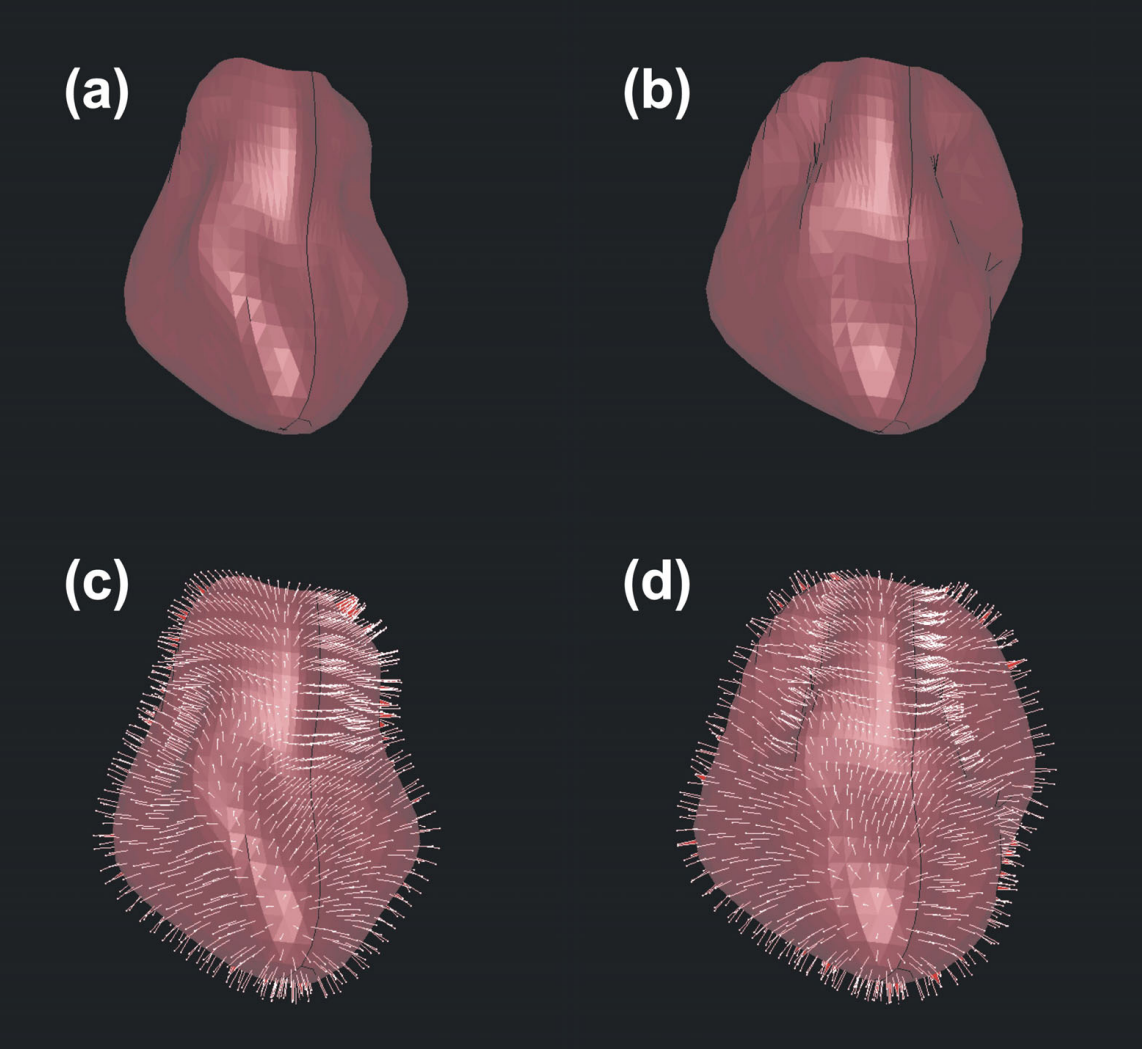
**Pressure direction at systole and diastole. **Rendered LV at systole on the left (a) and (c) and diastole on the right (b) and (d). Shown is the mesh generated with FEM program including all 774 FEM elements rendered with a standard constant shading model in (a) and (b). (c) and (d) show the mesh together with the surface vectors (normals) orthogonally placed on each element (triangle) indicating the pressure directions.

Both, the Philips Sonos 5500/7500 or the Acuson Sequoia ultrasound systems required less than 10 minutes acquistion time per patient. The application of the TomTec LV analysis algorithms with manual placement of the necessary landmarks took approximately 7 minutes per patient. In five patient data sets conversion from TomTec data to the FEM model was carried out in less than a minute for a heart cycle using the conversion tool MVCP FemCoGen^© ^[7]. ABAQUS processing time on the above computer was 20 seconds per sequence or approximately 6 minutes per patient. Total time for the procedure was approximately 24 minutes per patient (see Table). Fig. 3 shows the ABAQUS FEM program system interface (ABAQUS/viewer) including the LV models in default mesh mode. All 774 triangles of the FEM mesh from the diastolic state (14^th ^image out of a set of 18 images per heart cycle) are displayed and can be visualized using ABAQUS viewer options. Each mesh element can be analyzed separately. This is shown in Fig. 4: In Fig. 4a and 4b the rendered LV using a standard constant-shading model is displayed in systolic and diastolic states. Fig. 4c and 4d show both the FEM mesh and the normal vectors orthogonally placed (orthonormals) on each triangle indicating the force direction. These figures demonstrate the quantification of movement during the heart cycle directly using modeled continuous LV pressure and tissue elastance parameters.

**Table 1:** Data acquisition and processing times Data acquisition and processing times

Data set	Ultrasound acqusition [s]	TomTec analysis [s]	FemCoGen transfer [s]	Abaqus processing [s]
1	715	475	50	355
2	580	364	45	340
3	670	320	55	390
4	640	390	45	410
5	597	423	45	430
Mean ± (standard deviation) [s]	640.4 ± 41.7	394.4 ± 43.7	48 ± 3.6	385 ± 30
Total time [s]	1467.8 ± 29.7			

## Discussion

The general intention of this study was to demonstrate the feasibility of transporting individual patient's LV geometry data into a FEM model. Standard laptop computer technology was utilized to accomplish the transfer from common TEE-machines (Philips Sonos 5500/7500 and Acuson Sequoia).

The software running on the laptop was the commercially available TomTec LV Analysis TEE^© ^package and ABAQUS FEM system, plus the recently developed MVCP FemCoGen^©^. Accomplishing this transfer will form the foundation for intraoperative surgical planning and quantitative outcome assessment of valvular and LV reconstructive surgery.

The scope of this study was to produce a prototype in which the feasibility of the method could be assessed. In a fully operational system, we could postulate clinical applications such as enhanced/automated wall motion abnormality detection, assessment of regional relaxation which encompases the entire ventricle, assessment and guidance of ventricular remodeling operations, and serial assessment of recovery of regional wall function post myocardial stunning.

FEM meshes have been used for approximately 30 years [8] in the analysis of many anatomical structures and organs e.g such as major vessels [9,10], heart valves [11] and ventricles [12], lung [13], corneoscleral shell [14], plastic and reconstructive craniofacial surgery [15] and the femur [16]. A FEM model can be created to determine the deformation of the LV loaded by intraventricular pressure. Steady-state fluid dynamics and structural analyses can be carried out using commercial codes based on FEM [17]. At a sequence of time-steps of the cardiac cycle, the model can be considered to be a quasi-incompressible transversely isotropic hyperelastic material based on the analysis of Feng [18]. Until now, biomechanical cardiac FEM models have been based on simplified ellipsoidal and cylindrical geometries [18]. A FEM created in this way is not patient-specific and does not accurately represent precise regional deformations in the LV loaded by intraventricular pressure. The method described here will allow patient specifity and the precise representation of deformation. Our method would be applicable to the "live 3D" systems assuming that the entire ventricle could be seen throughout the cardiac cycle in the transthoracic (or epicardial) matrix array acquisition. This would be most feasible in small adults and children and can be proved in further studies.

The total time required for acquisition to a completed FEM model was approximately 24 minutes and can be accomplished during the time period when the patient is being prepared for cardiopulmonary bypass (generally 1 to 1.5 h). Thus the feasilbility in terms of duration is clearly demonstrated.

In terms of procedure accuracy, reproducibility and duration, the primary limitation is the dependence of the TomTec software on manual entries of the three registration landmarks. This requirement is iterative. Manual entries must be done for multiple frames within the TEE data sets. Inter- and intraobserver variability is a general problem for ultrasonic imaging. The validation of the TomTec border detection has not been published. TomTec LV Analysis TEE^© ^Software is under review by the US FDA, but despite of lack of validation TEE is the only practical technology in the cardiac operating room for the forseeable future. Ultrasound tissue Doppler technologies may be developed in the future to allow automation of the registration process.

A limitation of the present study is that it is focused on the deployment of the transfer method. The entire process will require extensive validation. The validation strategy will most likely involve comparision with preoperative cardiac MRI as well as comparison with bypass and post bypass tissue geometry in the same patients. Creating models from MRI based data sets analogous to the TomTec LV analysis and transfering these models to ABAQUS might lead to a new validation strategy which is not been possible up to now. The tool for modeling presented here facilitates vector-subtraction analysis for different points within the cardiac cycle. Quantification is therefore immediately available for both global and regional wall motion, shape and volume analysis. The future use of such instantaneous analysis has a number of potential applications for LV function assessment and surgical planning. This technology could enable a comprehensive automated regional wall motion analysis. A significant challenge in the evaluation and management of patients with coronary artery disease is determining the viability of myocardium. A biomechanical FEM of the LV myocardium can be imported to evaluate dynamic mechanical properties of regions of the myocardium. This approach could provide the basis for a new index of regional myocardial viability.

## Conclusions

For complete intraoperative 3D LV finite element analysis, three input elements are necessary: 1. time-gaited, reality-based structural information, 2. continuous LV pressure and 3. instantaneous tissue elastance. The first of these elements is now available using the methods presented herein. The later two parameters will be required for robust modeling and analysis. Pressure data will be easily available in the cardiac operating room. Strategies for computing elastance are presently under development. With all three parameters, it will be possible to begin to develop the computational strategies which will allow virtual procedures to be performed utilizing 3D display technology and a haptic-feedback robotic "instruments". Whether this new intraoperative information will be useful in assessing the effectiveness of surgical interventions such as LV remodeling remains to be studied.

FEM analysis has not been feasible for LV in the intraoperative setting. The major roadblock was the complexity and the practicality of transfer of structural 3D data to a FEM analysis program. This study describes a method to rapidly transfer 3D structural data from the TEE device into a FEM analysis program. Once mesured pressure and calculated elastance are added to the model, near real-time dynamic stress-strain information in the operating room will be achievable.

## Authors' contributions

JFV did the technical part implementing the FEM model in ABAQUS^®^, NSN did the data acquisition and the medical part. Both authors read and approved the final manuscript.
